# Glycosylation-related molecular subtypes and risk score of hepatocellular carcinoma: Novel insights to clinical decision-making

**DOI:** 10.3389/fendo.2022.1090324

**Published:** 2022-12-20

**Authors:** Yanlong Shi, Yizhu Wang, Rui Yang, Wenning Zhang, Yu Zhang, Kun Feng, Qingpeng Lv, Kaiyi Niu, Jiping Chen, Li Li, Yewei Zhang

**Affiliations:** ^1^ Hepatopancreatobiliary Center, The Second Affiliated Hospital of Nanjing Medical University, Nanjing, Jiangsu, China; ^2^ The Second Clinical Medical College, Lanzhou University, Lanzhou, Gansu, China; ^3^ Department of General Surgery, Fuyang Hospital of Anhui Medical University, Fuyang, Anhui, China

**Keywords:** glycosylation, metabolism, hepatocellular carcinoma, molecular subtype, immunotherapy, drug sensitivity, decision-making, consensus clustering

## Abstract

**Background:**

Hepatocellular carcinoma (HCC) is the fifth most common cancer and the third leading cause of cancer deaths worldwide, seriously affecting human community health and care. Emerging evidence has shown that aberrant glycosylation is associated with tumor progression and metastasis. However, the role of glycosylation-related genes in HCC has notbeen reported.

**Methods:**

Weighted gene coexpression network analysis and non-negative matrix factorization analysis were applied to identify functional modules and molecularm subtypes in HCC. The least absolute shrinkage and selection operator Cox regression was used to construct the glycosylation-related signature. The independent prognostic value of the risk model was confirmed and validated by systematic techniques, including principal component analysis, T-distributed random neighbor embedding analysis, Kaplan–Meier survival analysis, the ROC curve, multivariate Cox regression, the nomogram, and the calibration curve. The single-sample gene set enrichment analysis, gene set variation analysis, Gene Ontology, and Kyoto Encyclopedia of Genes and Genomes analyses were evaluated by the immune microenvironment and potential biological processes. The quantitative real-time polymerase chain reaction and immunohistochemistry analysis were used to verify the expression of five genes.

**Results:**

We identified the glycosylation-related genes with bioinformatics analysis to construct and validate a five-gene signature for the prognosis of HCC patients. Patients with HCC in the high-risk group had a worse prognosis. The risk score could be an independent factor and was associated with clinical features, such as the grade and stage. The nomogram exhibited an accurate score that included the risk score and clinical parameters. The infiltration levels of antitumor cells were upregulated in the low-risk group, including B_cells, Mast_cells, neutrophils, NK_cells, and T_helper_cells. Moreover, glycosylation was more sensitive to immunotherapy, and may play a critical role in the metabolic processes of HCC, such as bile acid metabolism and fatty acid metabolism. In addition, the five-gene messenger RNA (mRNA) and protein expression were overexpressed in HCC cells and tissues.

**Conclusions:**

The glycosylation-related signature is effective for prognostic recognition, immune efficacy evaluation, and substance metabolism in HCC, providing a novel insight for therapeutic target prediction and clinical decision-making.

## Introduction

Hepatocellular carcinoma (HCC) is the fifth most common cancer and the third leading cause of cancer deaths worldwide, seriously affecting human community health and care (1, 2). People with a history of chronic liver diseases, such as hepatitis B virus and alcoholic steatohepatitis, are more likely to progress to HCC. However, non-alcoholic fatty liver disease is rapidly becoming a dominant cause of HCC (3). Although there are many treatments for HCC, including surgery, chemotherapy, radiofrequency ablation, and liver transplantation, their efficacy is not always satisfactory (4). Due to the high heterogeneity of tumors, patients with HCC have a poor prognosis and high mortality, with the 5-year survival rate of 18% (5). It is necessary to excavate more effective prognostic biomarkers for therapeutic targets and clinical decisions.

Glycosylation is a complex form of protein modification in the biological process that inserts sugar chains into macromolecules such as proteins, DNA, and lipids, which directly lead to the mutation or inactivation of biological macromolecules (6, 7). Aberrant glycosylation, a hallmark of cancer, is a consequence and a driver of malignant phenotypes, directly impacting key processes supporting tumor progression and metastasis, including cell adhesion, motility, invasion, and immune evasion (8, 9). Sustained high glucose can promote abnormal glycosylation, activate specific signaling pathways, and produce irreversible toxic products, thereby accelerating HCC proliferation and metastasis (10, 11). Moreover, evidence suggested that the glycosylation-related genes were associated with Programmed cell death-Ligand 1 (PD-L1) expression and immune infiltration, which was helpful to investigate the diagnosis and targeted therapy in head and neck squamous cell carcinoma (12). However, the role of glycosylation-related genes in HCC has not been reported.

Herein, we identified a novel prognostic glycosylation-related signature using non-negative matrix factorization (NMF) and weighted gene coexpression network analysis (WGCNA) analysis followed by least absolute shrinkage and selection operator (LASSO) regression construction in HCC. Importantly, we verified the expression of five-signature genes by experiments in HCC. A systematic analysis of the model results, including the risk score, independent factors, immune microenvironment, functional enrichment, and drug sensitivity, may reference the association between glycosylation and HCC for further study.

## Methods

### Data acquisition

**Figure 1:**
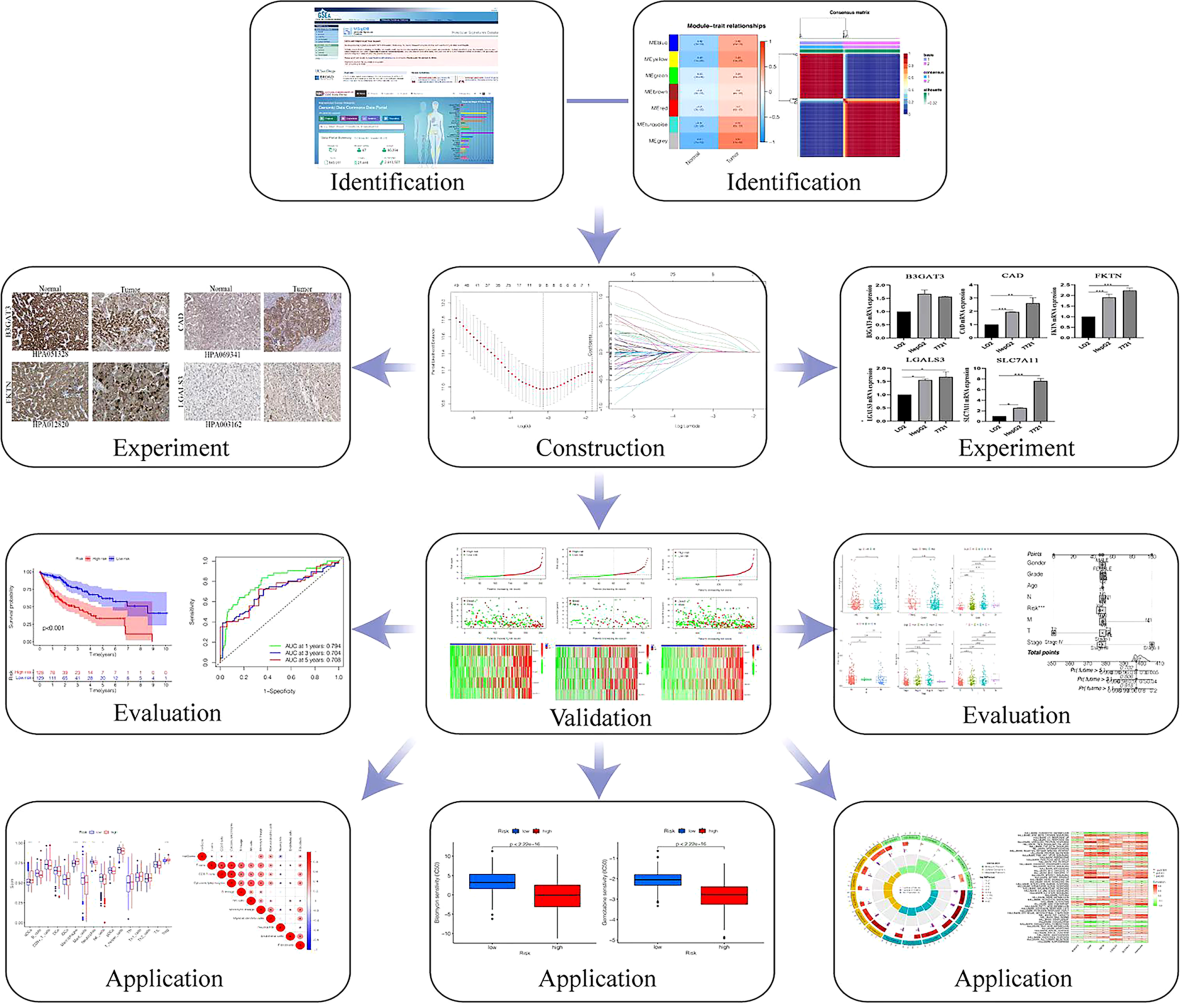
The flow chart of the study.

The overall outline of the study is presented in **Figure 1**. The RNA-sequencing data (365 HCC and 50 adjacent normal samples) and clinical information were downloaded from The Cancer Genome Atlas (TCGA, https://portal.gdc.com) (13). The expression profiles were normalized by log2 fragments per kilobase. The totals of glycosylation-related genes were obtained from gene set enrichment analysis (GSEA) (**Supplementary Table 1**).

### Identification of functional module and molecular subtype

Based on glycosylation-related genes, we extracted the expression profiles in the TCGA database and analyzed the differentially expressed genes (DEGs) by the R package “limma” with the values of FDRP< 0.05, |log2FC| > 1 in HCC. The WGCNA was applied to identify the strongest correlation module of glycosylation (14). Outlier samples were removed by hierarchical clustering analysis. By analyzing the appropriate soft threshold power, a scale-free network is established. The clustering of coexpression modules is based on the dynamic tree-cutting method. The glycosylation-related DEGs were detected in HCC-related modules, and their correlation with module membership was analyzed. Finally, we selected the most robust correlation module as candidate genes for further study. Moreover, the candidate genes were used for NMF analysis with the “brunet” standard and 50 iterations. Moreover, the value of k defined as 2–10. The optimal k was dependent on the indexes of cophenetic, dispersion, and silhouette (15). The Kaplan–Meier (K-M) curves were used to show overall survival (OS) and progression-free survival (PFS) in two clusters.

### Construction and estimation of glycosylation-related signature

LASSO Cox regression analysis was used to construct prognostic signature by R package “glmnet.” To avoid overfitting, we also introduced a penalty parameter (λ) to risk model by 10-fold cross-validations (16). The formula of risk score was as follows: $\text{risk score}=\sum_{i=1}^{n}\left(\text{Expi}*\text{Coei}\right)$ . The five glycosylation-related genes and corresponding coefficients were identified in the prognostic model. Then, patients were divided into training and testing cohorts. According to the median cutoff value, each cohort was classified into low- and high-risk groups. The PCA and t-SNE analysis aimed to evaluate the ability to distinguish classification of two risk groups (17). Moreover, the R packages “plot,” “pheatmap,” “survival,” and “timeROC” were applied to evaluate the status, survival, and ROC in the training, testing, and total cohorts.

### Independent prognostic signature validation

Univariate and multivariate Cox regression analyses were conducted to determine the relationship between the risk score and clinical features, and the specificity and sensitivity of risk score were investigated for 1, 3, and 5 years by the ROC curve. To forecast survival probability, we established the nomogram containing the risk score and clinical features by the R package “rms” (18). Then, the calibration curve and ROC curves verified the consistency between actual survival time and probability OS in 1, 3, and 5 years. To further identify independent prognostic factors, the R package “heatmap” and K-M curve analysis were used to detect critical features among age, gender, grade, T stage, N stage, and M stage in low- and high-risk groups.

### Evaluation of tumor infiltration and immune response

The single-sample GSEA was conducted to perform the association between risk groups and immune cells, and immune functions, with parameters as follows: kcdf= ‘Gaussian’, method=ssgsea, and ranking=TRUE (19). The correlation coefficients between risk scores and immune infiltration cells and immune checkpoints were presented in a heatmap. Then, the composition of 22 infiltrating immune cells was established by R package “CIBERSORT” in different risk groups (20). The Wilcoxon rank-sum test analyzed the expression of immune checkpoints in risk groups.

### Functional enrichment analysis

The Gene Ontology (GO) and Kyoto Encyclopedia of Genes and Genomes (KEGG) pathway functional enrichment were used to explore potential biological functions between low- and high-risk groups through R package “cluster Profiler.” The hallmarks and KEGG of gene set variation analysis (GSVA) were also selected (21), with the threshold value as follows: Permutations: 1000 times, and P**<**0.05.

### Prediction of drug response

The genomics of drug sensitivity in the cancer database was used to predict the responses to some chemotherapy drugs between low- and high-risk groups through R package “pRRophetic,” with the threshold of half-maximal inhibitory concentration (IC50) (22).

### Cell culture

The normal hepatic cell (LO2) and HCC cell line (HepG2 and 7721), were donated from the First Hospital Affiliated to Anhui Medical University. Furthermore, the DMEM with high-glucose (HyClone) and 10% fetal bovine serum (VivaCell, Shanghai, China) were treated to these cells in 5% CO_2_, 37°C.

### Quantitative real-time polymerase chain reaction

Total RNA was extracted from different cells through the TRIzol reagent. The complementary DNA (cDNA) was obtained from reverse transcription using PrimeScript™ kit. Then, as a quantitative reagent, the SYBR Green qPCR Mix was applied to test the gene expression of the prognostic signature. The results were calculated as the 2^−ΔΔCt^ method. All primer sequences are illustrated in **Supplementary Table 2**.

### Analysis of immunohistochemistry

The expression of protein was detected by immunohistochemistry in normal and HCC tissues. In the HPA database, we explored the image of 5-glycosylation-related genes protein expression in “tissue” and “pathology” of modules (23). Patients and the images of serial numbers are included in this research. All images were rejudged by two pathologists. Regents are as follows: B3GAT3: Atlas Antibodies Cat#HPA051328, RRID : AB_2681444, dilution: 1:130; CAD: Atlas Antibodies Cat#HPA069341, RRID : AB_2686125, dilution: 1:500; Atlas Antibodies Cat#HPA012820, RRID : AB_1848478, dilution: 1:25; Atlas Antibodies Cat#HPA003162, RRID : AB_1078937, dilution: 1:15.

### Statistical analysis

The R software (version 4.1.2) was used for statistical analyses and visualization in this research. Qualitative data are expressed as percentages. The t test or ANOVA analysis was used for normally distributed data of two or more groups, and the chi-square test or Fisher’s test was used for other data. The differences in survival between risk groups were conducted by K-M analysis with a log-rank test. The ROC curve was used to evaluate the efficiency of the signature. *P*< 0.05 was defined as statistically significant.

## Results

### Identification of glycosylation-related genes, functional modules, and molecular subtypes

**Figure 2:**
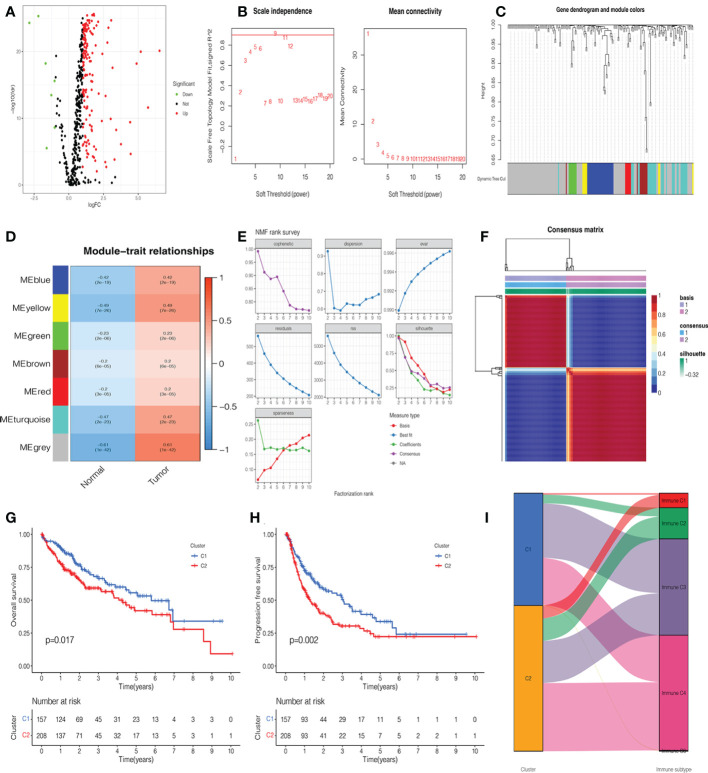
Identification of glycosylation-related differentially expressed genes (DEGs), functional modules, and molecular subtypes. **(A)** Volcano plot of 201 glycosylation-related DEGs in hepatocellular carcinoma (HCC). **(B)** The distribution of the scale-free topology model fit and the trends of mean connectivity. **(C)** The hierarchical clustering analysis presented similar characteristics with the same color by a dendrogram. **(D)** The correlation between the trait and each module in HCC and normal tissues. **(E)** Non-negative matrix factorization (NMF) survey analyzed the factorization rank, including cophenetic, dispersion, evar, residuals, rss, silhouette, and sparseness coefficients. **(F)** Heatmap of two clusters of HCC. Kaplan–Meier (K-M) curves of overall survival (OS) **(G)** and progression-free survival (PFS) **(H)** in two clusters of patients with HCC. **(I)** The association between clusters and immune C1, C2, C3, and C4.

A total of 365 patients with HCC who had follow-up data were included in the study. Combined with glycosylation-related genes, we identified DEGs in the TCGA cohort, including 152 upregulated genes and 7 downregulated genes. The volcano map and heatmap visualize the differences between HCC tissues and adjacent non-tumor tissues (**Figure 2A**, **Supplementary Figure 1A**). To investigate the functional modules of glycosylation-related genes in HCC, we first conducted clustering dendrograms to detect the outliers of 365 HCC and 50 normal tissues by WGCNA. There were no outliers among these tissues (**Supplementary Figure 1B**). The weighted value β was scheduled as 9, which emerged as a good consistency in a scale-free network (**Figure 2B**, **Supplementary Figure 1C**). We identified seven functional modules, including the blue module (27 genes), yellow module (8 genes), green module (8 genes), brown module (10 genes), red module (6 genes), turquoise module (31 genes), and gray module (101 genes) (**Figure 2C**, **Supplementary Figure 1D**). Among them, it was found that the gray module had the strongest correlation between normal and HCC tumors (r = 0.61, p = 1e-42) (**Figure 2D**). Therefore, the 101 genes were used as candidate genes for further analysis (**Supplementary Table 3**).

NMF analysis was applied to determine the optimal molecular subtype of 101 glycosylation-related genes in HCC. According to the results of the NMF rank survey, the optimal k value was identified as 2 (**Figure 2E**). The corresponding heatmap suggested a definite boundary than others (**Figure 2F**, **Supplementary Figure 1E**). Moreover, cluster 1 had a good OS (P=0.017) (**Figure 2G**) and PFS (P=0.002) (**Figure 2H**) than cluster 2 in patients with HCC. In **Figure 2I**, cluster 1 was associated with immune C3, but cluster 2 was associated with immune C4.

### Analysis of independent prognostic value and construction of prognostic nomogram

**Figure 3:**
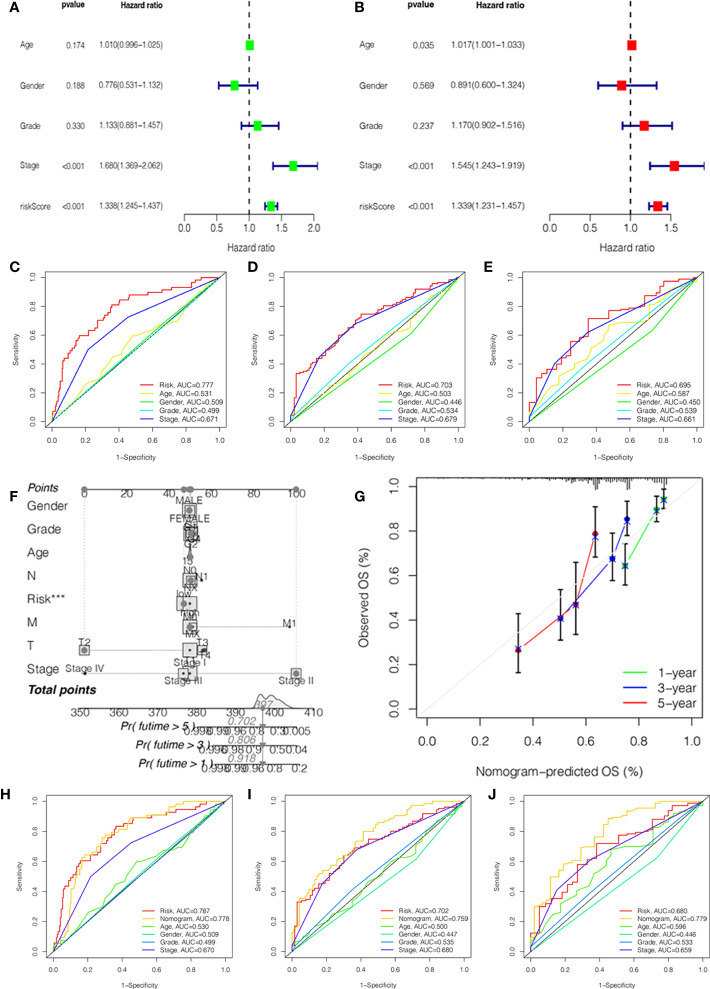
Independent prognostic value validation. **(A)** The relationship between the risk score and clinicopathological features by univariate Cox analysis. **(B)** The relationship between the risk score and clinicopathological features by multivariate Cox analysis. The AUC values of the ROC curve of 1 **(C)**, 3 **(D)**, and 5 years **(E)** for the risk score and clinicopathological features. **(F)** Nomogram for 1-, 3-, and 5-year OS prediction. **(G)** Calibration curves for 1-, 3-, and 5-year OS prediction. **(H-J)** The AUC values of the ROC curve of 1, 3, and 5 years for the nomogram.

To investigate the independent prognostic factors, we first analyzed the clinical features by univariate and multivariate Cox regression. The results suggested that the stage and risk score of HCC patients had a higher hazard ratio with 95% confidence interval (P<0.001) (**Figures 3A, B**). Then, combined with the clinical features, we further explored the ROC curves of the stage and risk score. In 1, 3, and 5 years, the area under the curve (AUC) values of HCC patients were 0.777, 0.703, and 0.695 of the risk score and 0.671, 0.679, and 0.661 of the stage (**Figures 3C–E**).

In order to better predict the survival probability of HCC patients, we made an attempt to develop a clinical application tool. The OS probability of the nomogram in 1, 3, and 5 years was 0.918, 0.806, and 0.702, respectively (**Figure 3F**). The calibration curve indicated that the nomogram had remarkable prediction performance and stability (**Figure 3G**). Afterward, The ROC curves of nomogram identified the AUC for 1-, 3-, and 5-year HCC patients as 0.778, 0.759, and 0.779, respectively (**Figures 3H–J**). Therefore, the risk score and stage were independent factors, and the nomogram could be a reliable nomogram for survival prediction.

**Figure 4:**
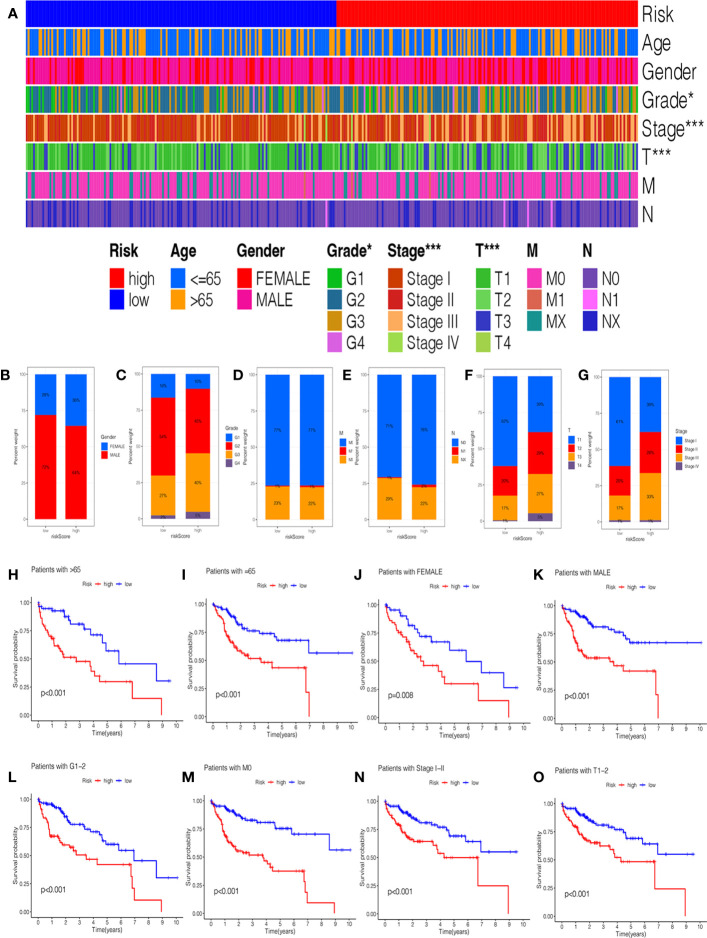
The correlation of clinicopathological features with the prognostic signature. **(A)** The heatmap analysis between clinicopathological characteristics and low- and high-risk groups. The proportion of different clinicopathological characteristics in low- and high-risk groups: **(B)** Gender. **(C)** Grade. **(D)** M stage. **(E)** N stage. **(F)** T stage. **(G)** stage. The K-M survival analysis of clinicopathological factors between low- and high-risk groups: **(H)** Age ≥ 65. **(I)** Age = 65. **(J)** Female. **(K)** Male. **(L)** Grade 1–2. **(M)** M0 stage. **(N)** Stage I-II. **(O)** T1–2 stage.

In addition, we investigated the relationship between the risk score and clinical features, and the results suggested no significant difference in the age, gender, grade, M stage, and N stage except for the stage and T stage (**Supplementary Figures 2A–F**). The heatmap manifested the association between risk groups and clinical features, including the grade, stage, and T stage (**Figure 4A**). Furthermore, the proportion of different clinical features in low- and high-risk groups is presented in **Figures 4B–G**. Interestingly, the K-M curve demonstrated that patients in the low-risk group had a longer survival probability than those in low-risk group under the conditions of female, man, age > 65, age ≤ 65, G1–G2, G3–G4, M0, N0, Nx, stage I–II, stage III–IV, T1–T2, and T3–T4 (**Figures 4H-O**, **Supplementary Figures 2G–L**).

### Identification and validation of glycosylation-related signature in patients with HCC

**Figure 5:**
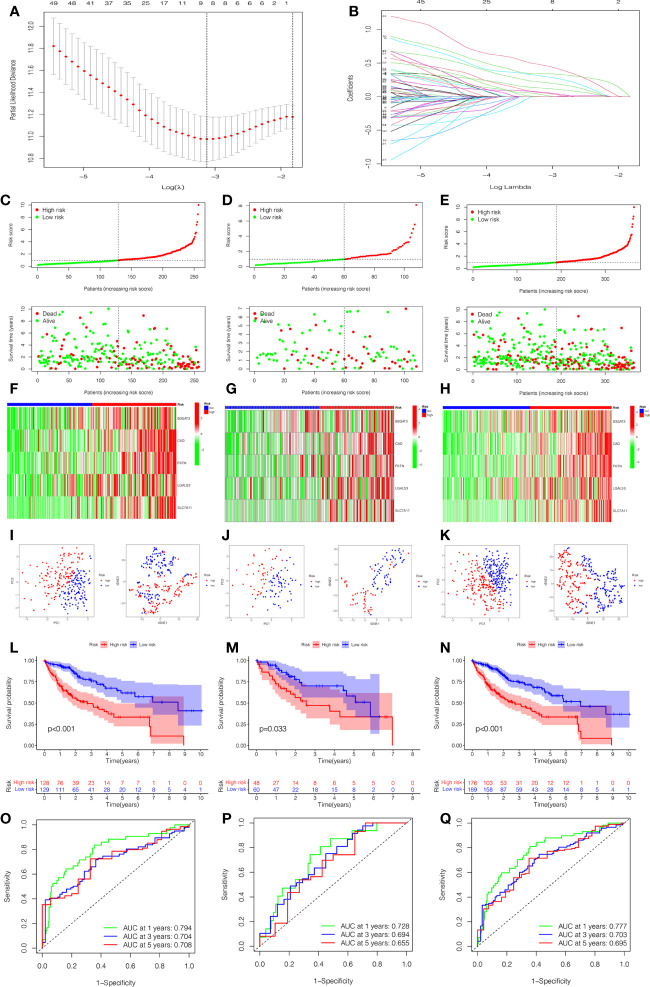
Construction and estimation of the glycosylation-related signature. **(A)** LASSO coefficient distribution of the glycosylation-related signature. **(B)** The optimal parameter (λ) selection by the cross-validation curve. The distribution and survival status of the risk score in the training cohort **(C)**, testing cohort **(D)**, and total cohort **(E)**. Heatmap of five-gene expression between low- and high-risk groups in the training cohort **(F)**, testing cohort **(G)**, and total cohort **(H)**. The PCA and t-SNE analysis in the training cohort **(I)**, testing cohort **(J)**, and total cohort **(K)**. **(L–N)** The K-M survival analysis between low- and high-risk groups in the training cohort **(L)**, testing cohort **(M)**, and total cohort **(N)**. The AUC values of ROC curves for 1, 3, and 5 years in the model in the training cohort **(O)**, testing cohort **(P)**, and total cohort **(Q)**.

To quantify the prognosis of each patient, the 101 of candidate genes were used for the glycosylation-related signature by LASSO regression with the optimal regression coefficient and 10-fold cross-validation (**Figures 5A, B**). The risk score for the signature was as follows: risk score= 0.546 × expression of B3GAT3 + 0.412 × expression of CAD + 0.704 × expression of FKTN + 0.202 × expression of LGALS3 + 0.349 × expression of SLC7A11. Then, a total of 365 patients with HCC were randomly classified into training and testing cohorts. After excluding patients with unknown clinical information, we presented the differences in clinicopathological characteristics between training and testing cohorts (**Table 1**). According to the median score, the patients with HCC were divided into low- and high-risk groups. Subsequently, the scatterplot, risk curve, and risk heatmap were applied to show risk score distribution, the survival status, and the expression between low- and high-risk groups in the training, testing, and total cohorts (**Figures 5C–H**). It was founded that the patients in the high-risk group had higher risk coefficients and mortality. PCA and t-SNE analysis further verified that the risk score model had good discrimination performance in training, testing, and total cohorts (**Figures 5I–K**). To assess the predictive quality and accuracy of the signature, the K-M survival curve was used to the predictive ability of signature, and the results revealed that patients in the high-risk group had a poorer OS than those in the low-risk group in the training cohort (P< 0.001) (**Figure 5L**). This result was consistent with the testing cohort (P<0.05) (**Figure 5M**) and total cohort (P< 0.001) (**Figure 5N**). Moreover, in the 1-, 3-, and 5-year follow-ups, the AUC values of ROC curves were 0.794, 0.704, and 0.708 of the training cohort (**Figure 5O**); 0.728, 0.694, and 0.655 of the testing cohort (**Figure 5P**); and 0.777, 0.703, and 0.695 of the total cohort (**Figure 5Q**), respectively.

**Table 1:** The Clinicopathological Characteristics in Training and Testing Cohort.

Characteristics	Total (%)	Training cohort(%)	Testing cohort(%)	P value
Age				
<=65	218 (64.69)	151 (63.98)	67 (66.34)	0.77
>65	119 (35.31)	85 (36.02)	34 (33.66)	
Gender				
FEMALE	107 (31.75)	72 (30.51)	35 (34.65)	0.53
MALE	230 (68.25)	164 (69.49)	66 (65.35)	
Grade				
G1	45 (13.35)	32 (13.56)	13 (12.87)	0.18
G2	166 (49.26)	109 (46.19)	57 (56.44)	
G3	114 (33.83)	84 (35.59)	30 (29.7)	
G4	12 (3.56)	11 (4.66)	1 (0.99)	
Stage				
Stage I	168 (49.85)	120 (50.85)	48 (47.52)	0.12
Stage II	82 (24.33)	61 (25.85)	21 (20.79)	
Stage III	83 (24.63)	54 (22.88)	29 (28.71)	
Stage IV	4 (1.19)	1 (0.42)	3 (2.97)	
T				
T1	170 (50.45)	122 (51.69)	48 (47.52)	0.03
T2	83 (24.63)	62 (26.27)	21 (20.79)	
T3	74 (21.96)	49 (20.76)	25 (24.75)	
T4	10 (2.97)	3 (1.27)	7 (6.93)	
M				
M0	258 (76.56)	181 (76.69)	77 (76.24)	0.03
M1	3 (0.89)	0 (0)	3 (2.97)	
MX	76 (22.55)	55 (23.31)	21 (20.79)	
N				
N0	247 (73.29)	175 (74.15)	72 (71.29)	0.82
N1	4 (1.19)	3 (1.27)	1 (0.99)	
NX	86 (25.52)	58 (24.58)	28 (27.72)	

### Correlation of immune infiltration and immunotherapy response with prognostic signature

**Figure 6:**
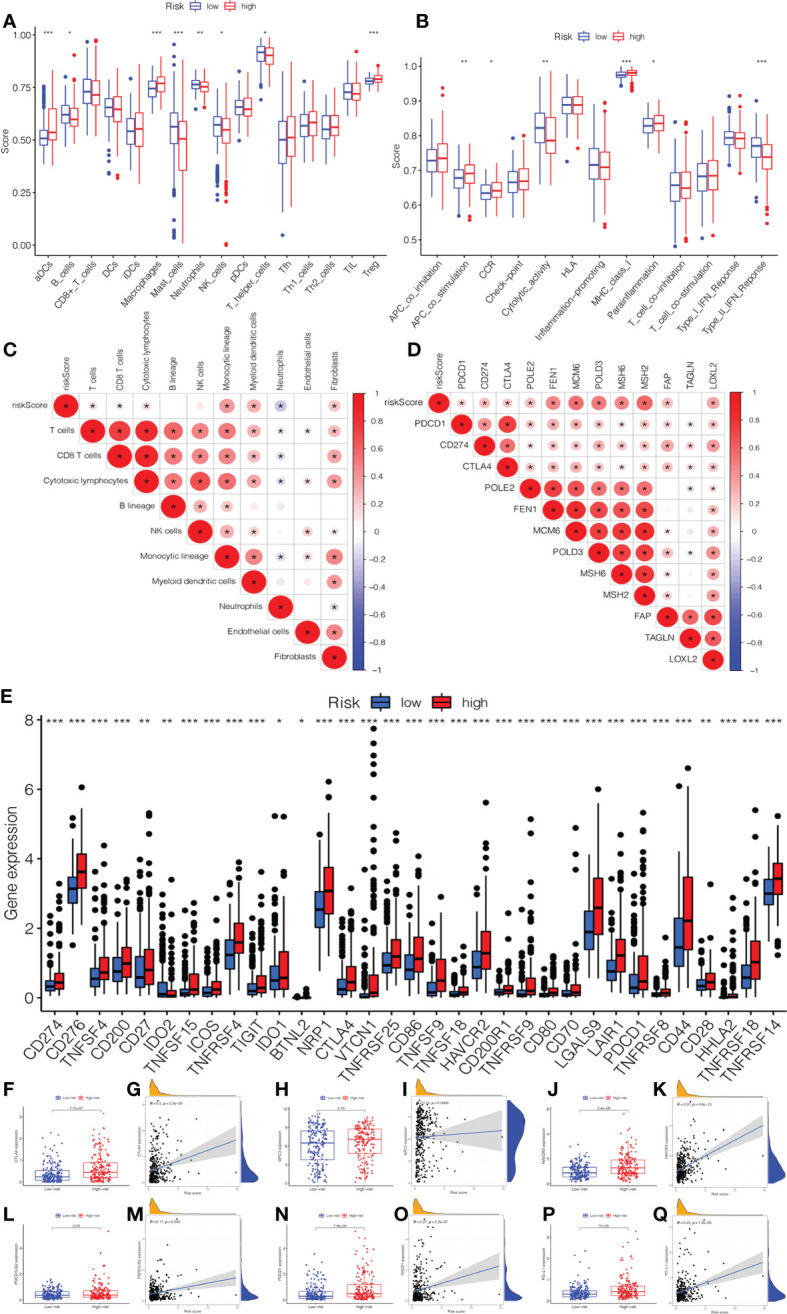
The association of immune infiltration and immunotherapy response with the prognostic signature. **(A)** The infiltrating levels of 16 subtypes of immune cells in low- and high-risk groups. **(B)** The expression of 13 immune functions in low- and high-risk groups. **(C)** The correlation between the risk score and immune infiltration cells. **(D)** The expression of the immune checkpoints of low- and high-risk groups. **(E)** The correlation between the risk score and immune checkpoint genes. The expression and correlation between the risk score and immune checkpoint inhibitors: **(F)** CTLA4. **(G)** GPC3. **(H)** HAVCR2. **(I)** PDCD1LG2. **(J)** PDCD1. **(K)** PD-L1.

In **Figures 6A, B**, we compared the differences in immune cells and immune functions between the low- and high-risk groups. The score of B_cells, Mast_cells, neutrophils, NK_cells, T_helper_cells, cytolytic_activity, and Type_II_IFN_Response in the low-risk group were significantly higher than those in the high-risk group. In addition, the activity of aDCs, Macrophages, Treg, APC_co_stimulation, MHC_Class_I, and Parainflammation markedly increased in the high-risk group. The CIBERSORT algorithm demonstrated a strong correlation between the risk score and immune infiltration cells (**Figure 6C**). Then, the bar plot exhibited the percentage of 22 types of immune infiltrating cells in the low- and high-risk groups (**Supplementary Figure 3A**). As shown in **Figure 6D**, we further explored the correlation between the risk score and immune genes. Moreover, it was found that the expression of immune checkpoints was significant between low- and high-risk groups (**Figure 6E**). In addition, we investigated in detail the expression of immune checkpoint inhibitors in low- and high-risk groups and the correlation between the risk score and immune checkpoints (CTLA4, GPC3, HAVCR2, PDCD1, PDCD1LG2, and PDL1). The expression of CTLA4, HAVCR2, PDCD1, and PDL1 in the high-risk group was higher than those in the low-risk group (**Figures 6F–Q** **)**. There was also a significant correlation between the risk score and immune checkpoints.

### Analysis of functional enrichment

**Figure 7:**
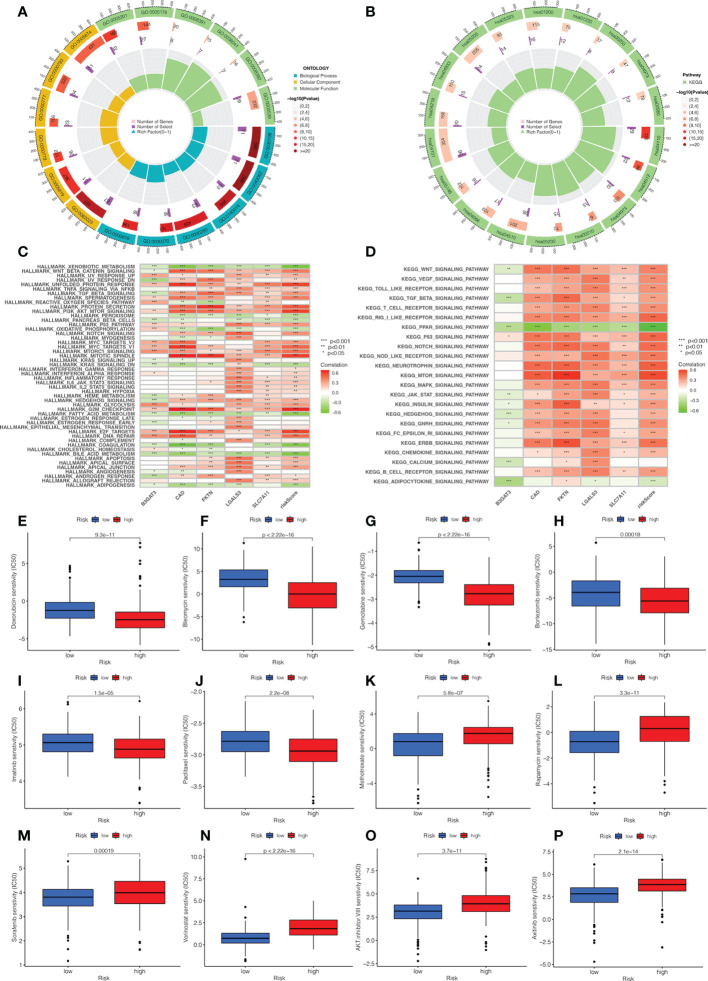
Analysis of functional enrichment and application of the signature in drug sensitivity. **(A)** The GO analysis by a chordal graph. **(B)** The KEGG analysis between low- and high-risk groups by a chordal graph. **(C)** The hallmark processes by GSVA analysis in five genes by GSVA analysis. **(D)** The KEGG signaling pathway by GSVA analysis. The association between drug sensitivity and low- and high-risk groups: **(E)** Doxorubicin. **(F)** Bleomycin. **(G)** Gemcitabine. **(H)** Bortezomib. **(I)** Imatinib. **(J)** Paclitaxel. **(K)** Methotrexate. **(L)** Rapamycin. **(M)** Sorafenib. **(N)** Vorinostat. **(O)** AKT.inhibitor VIII. **(P)** Axitinib.

We explored the biological functions and enriched pathways between low- and high-risk groups. The two risk groups may be involved in cellular division and glycosaminoglycan binding by GO analysis (**Figure 7A**, **Supplementary Figure 3B** **)**. KEGG analysis indicated that the top three pathways were human papillomavirus infection, the PI3k-Akt signaling pathway, and the cell cycle (**Figure 7B**, **Supplementary Figure 3C** **)**. To further determine the biological behaviors, GSVA was conducted to identify the hallmark process and KEGG pathway based on the risk score. Through GSVA, the signature genes were mainly enriched in xenobiotic metabolism, unfolded protein response, PI3k Akt MTOR signaling, KRAS signaling, hedgehog signaling, G2M checkpoint, E2F target, and bile acid metabolism (**Figures 7C, D** **)**. The KEGG of GSVA suggested that the risk score was positively correlated with the Wnt signaling pathway, VEGF signaling pathway, MAPK signaling pathway, and node-like receptor signaling pathway, while the risk score was negatively correlated with the PPAR signaling pathway.

### Application of the signature in drug sensitivity

In order to investigate the relationship between the risk score and clinical chemotherapy, drug sensitivity analysis was used to determine the clinical benefits of the signature for HCC. The IC50 values of drugs in the low-risk group were higher than those in the high-risk group, including doxorubicin, bleomycin, gemcitabine, bortezomib, imatinib, and paclitaxel. However, methotrexate, rapamycin, sorafenib, vorinostat, AKT.inhibitor.VIII, and axitinib were more sensitive to patients in the high-risk group (**Figures 7E–P**). These results could be a project for chemotherapy in patients with different risk groups.

### Verification of signature gene by quantitative real-time polymerase chain reaction and immunohistochemistry

**Figure 8:**
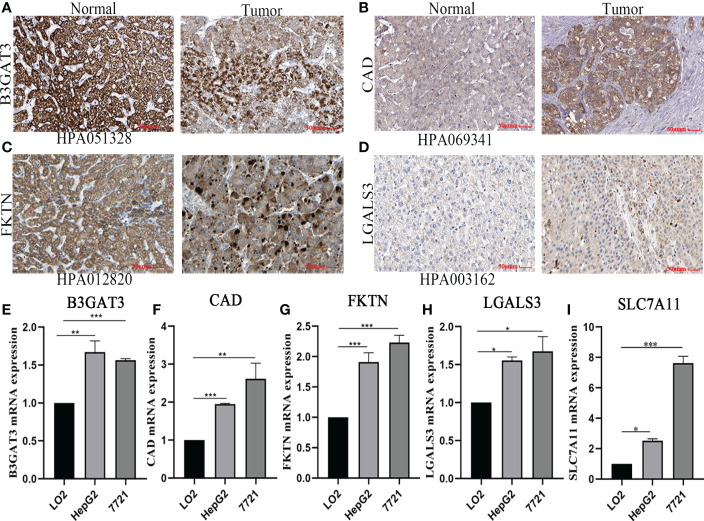
Verified protein and mRNA expression of the five-gene signature. Protein expression in HCC and normal tissues validated by the immunohistochemistry analysis of the HPA database: **(A)** B3GAT3. **(B)** CAD. **(C)** FKTN. **(D)** LGALS3. The expression of five-gene mRNA in hepatic and HCC cell lines experimented by quantitative real-time polymerase chain reaction analysis: **(E)** B3GAT3. **(F)** CAD. **(G)** FKTN. **(H)** LGALS3. **(I)** SLC7A11.

Based on the five-gene signature, we first detected the mRNA protein expression of five genes in HCC cell lines by quantitative real-time polymerase chain reaction (qRT-PCR) and immunohistochemistry analysis. Compared to normal tissues, the protein expression of B3GAT3, CAD, FKTN, and LGALS3 was positive in HCC tissues, mainly located in cytoplasmic/membranous (**Figures 8A–D**). However, the protein expression of SLC7A11 was not retrieved, and further studies are needed. Furthermore, we identified that the mRNA expression of B3GAT3, CAD, FKTN, LGALS3, and SLC7A11 were upregulated in HepG2 and 7721 cell lines (**Figures 8E–I**).

## Discussion

In this study, we screened 101 candidate genes and identified two subtypes of glycosylation in HCC patients. Based on these genes, we constructed a glycosylation-related signature and aimed to provide an individualized clinical diagnosis and treatment strategy for HCC patients. The prognostic value of the risk model was confirmed and validated by systematic techniques including PCA, t-SNE, K-M survival analysis, the ROC curve, and multivariate Cox regression. Our findings suggested that the risk score could be an independent factor and associated with clinical features, hence performing stability and predictability in the prognosis of patients with HCC. Moreover, the nomogram exhibited an accurate score that included the risk score and clinical parameters. The nomogram presents a score that includes risk scores and clinical parameters to guide clinicians in making individualized decisions.

The signature consisted of five glycosylation-related genes: B3GAT3, CAD, FKTN, LGALS3, and SLC7A11. B3GAT3 is a glycosyltransferase that plays a decisive role in proteoglycan synthesis (24). The abnormal expression of B3GAT3 accelerated the glycolytic pathway and promoted the proliferation of colorectal cancer cells, thereby affecting the prognosis of patients (25). The knockdown of B3GAT3 reversed the levels of epithelial–mesenchymal transition markers in HCC cells, which could be a novel prognostic biomarker for HCC (26). CAD is a multifunctional enzyme complex whose overactivation was associated with tumors primarily through metabolic programming and chemotherapy resistance (27, 28). CAD-induced pyrimidine synthesis and ribosome production promote the rapid recall reaction of memory T cells (29). FKTN expression was correlated with carcinogenesis and may be a key regulator of intestinal gastric cancer progression (30). A study in HCC indicated that LGALS3 expression was related to metastasis-related processes (31). Zhang et al. demonstrated that LGALS3 overexpression may involve recurrence and microenvironments in HCC (32). Moreover, SLC7A11 is a suppressor of ferroptosis, and its overexpression is associated with a poor prognosis in various cancers (33). SLC7A11-induced ferroptosis can be inhibited by SHP-1/STAT3-mediated MCL1 downregulation and BECN1 binding increase in HCC (34). These genes in our model were correlated with occurrence, recurrence, progression, and prognoses in HCC. In addition, our experimental results verified that the expression of five genes was upregulated in HCC cell lines by qRT-PCR analysis. Immunohistochemical analysis showed that these proteins expressions were positive in HCC tissues compared to adjacent normal tissues. Therefore, these results further demonstrated that the signature shows a superior predictive performance in HCC.

The phenotype and function of tumor cells can be disrupted by effective immune responses in the tumor microenvironment. The glycosylation process has been correlated with the tumor microenvironment further to determine the association between immune infiltration and risk groups. We acquired many differences between low- and high-risk groups in immune cell infiltration by the glycosylation-related gene signature. We found that the infiltration levels of antitumor cells were upregulated in the low-risk group, including B_cells, Mast_cells, neutrophils, NK_cells, and T_helper_cells. An important result of our study is that the expression of macrophages and Tregs was more abundant in the high-risk group. Accumulating evidence reported that increased levels of Tregs and macrophages had a worse prognosis for patients with HCC (35, 36). Moreover, the expression of Type_II_IFN_Response in the high-risk group was significantly higher than those in the low-risk group, which was identified as a key factor in coordinating the interaction between tumors and the immune system (37). Previous studies also demonstrated that the level of NK cells is positively correlated with the survival of patients with HCC (38, 39). Consistently our GSEA analysis suggested the infiltrating level of NK cells was increased in the low-risk group. Immune checkpoint inhibitors show new promise in antitumor therapy, mainly by blocking CTLA-4, GPC3, PDCD1, and PDL1 to enhance T-cell activity (40). Our study found that the expression of CTLA4, HAVCR2, PDCD1, and PDL1 was positively correlated with high-risk groups. This suggested that patients with high immune checkpoint inhibitor expression may be effective for immunotherapy. It was reported that CTLA-4 played a key role in maintaining self-tolerance and Treg suppression in HCC immunity (41). Moreover, PD-L1 could be not only an important mediator but also a critical target for antitumor therapy in HCC (42). These results confirmed that patients in the low-risk group were more sensitive to immunotherapy, which was consistent with the active tumor immune microenvironment and the high expression of immune checkpoints. Thus, the signature could accurately evaluate the tumor immune microenvironment and predict immune checkpoint inhibitor efficacy.

Additional analysis of functional enrichment suggested that the five-signature genes may be involved in the glucose metabolic process and cell cycle regulation, such as cellular division, glycosaminoglycan binding, and steroid hydroxylase activity. These pathways are in line with the glycosylation process, in which proteins or lipids are added to sugars. Through GSEA analysis, we identified that the five-signature genes were negatively correlated with xenobiotic metabolism, bile acid metabolism, fatty acid metabolism, and the PPAR signaling pathway but positively correlated with the unfolded protein response, G2M checkpoint, E2F target, PI3k Akt MTOR signaling, and P53 pathway. The metabolism of HCC could be altered by the inherent glycosylation characteristics (9). In addition, by performing drug sensitivity analysis, our study found that sorafenib was more sensitive to patients in the high-risk group, while doxorubicin was more sensitive in the low-risk group. The results confirmed the efficacy of sorafenib and lenvatinib in patients with unresectable HCC (43). Therefore, glycosylation may be involved in developing resistance, and more studies are needed to explain the underlying metabolic processes.

There are several limitations being addressed in this study. Firstly, although we identified prognostic genes through NMF and WGCNA models, the data of HCC were taken only from the TCGA database. Secondly, this is a retrospective study, and more multicenter, prospective studies are needed to verify the stability and accuracy of the signature in the future. Thirdly, the molecular mechanism of glycosylation-related genes needs to be further explored in HCC.

## Conclusion

We integrated glycosylation-related genes with bioinformatics analysis to construct and validate a five-gene signature for the prognosis of HCC patients. Our study demonstrated that the signature is effective for HCC prognostic recognition, immunotherapy response, and substance metabolism in HCC. Future studies should further elucidate the underlying mechanisms by which the five-gene signature regulates the immune microenvironment and provides a basis for immunotherapeutic strategies in HCC.

## Data availability statement

The original contributions presented in the study are included in the article/**Supplementary Materials**. Further inquiries can be directed to the corresponding authors.

## Author contributions

YS and YW conceived the study conception and design. YS, YW, RY, WZ and YZ wrote the manuscript. KF, QL and JC performed the experiments and analyzed the data. YZ and KN were responsible for the tables and figures. YS, YW and LL revised the manuscript. YWZ conceived and funded the study. All authors contributed to the article and approved the submitted version.

## Supplementary material

Supplementary Figure 1
**(A)** The heatmap of glycosylation-related DEGs. **(B)** Clustering dendrograms and trait heatmap of HCC and adjacent tissues. **(C)** The glycosylation-related DEGs clustering based on TOM dissimilarity. **(D)** All heatmaps obtained by NMF consensus clustering.

Supplementary Figure 2 Correlation between clinicopathological features and risk score or risk groups. Expression of clinicopathological features in risk score: **(A)** Age **(B)** Gender. **(C)** Grade. **(D)** M stage. **(E)** Stage. **(F)** T stage. Association between clinicopathological features and low- and high-risk groups by K-M survival analysis: **(G)** Grade 3-4. **(H)** M1 stage. **(I)** N0 stage. **(J)** Nx stage. **(K)** Stage III-IV. **(L)** T3-T4 stage.

Supplementary Figure 3 Analysis the proportion of immune infiltration cells and functional enrichment. **(A)** The proportion of immune infiltration cell in low- and high-risk groups. **(B)** The biological functions, cellular component, and molecular function between low- and high-risk groups. **(C)** The mainly signaling pathway by KEGG analysis.
