# Supplementary figures and images for: Glycosylation-related molecular subtypes and risk score of hepatocellular carcinoma: Novel insights to clinical decision-making
Source: Front Endocrinol (Lausanne). 2022 Dec 20;13:1090324. doi: 10.3389/fendo.2022.1090324 (PMC9807760; doi:10.3389/fendo.2022.1090324)

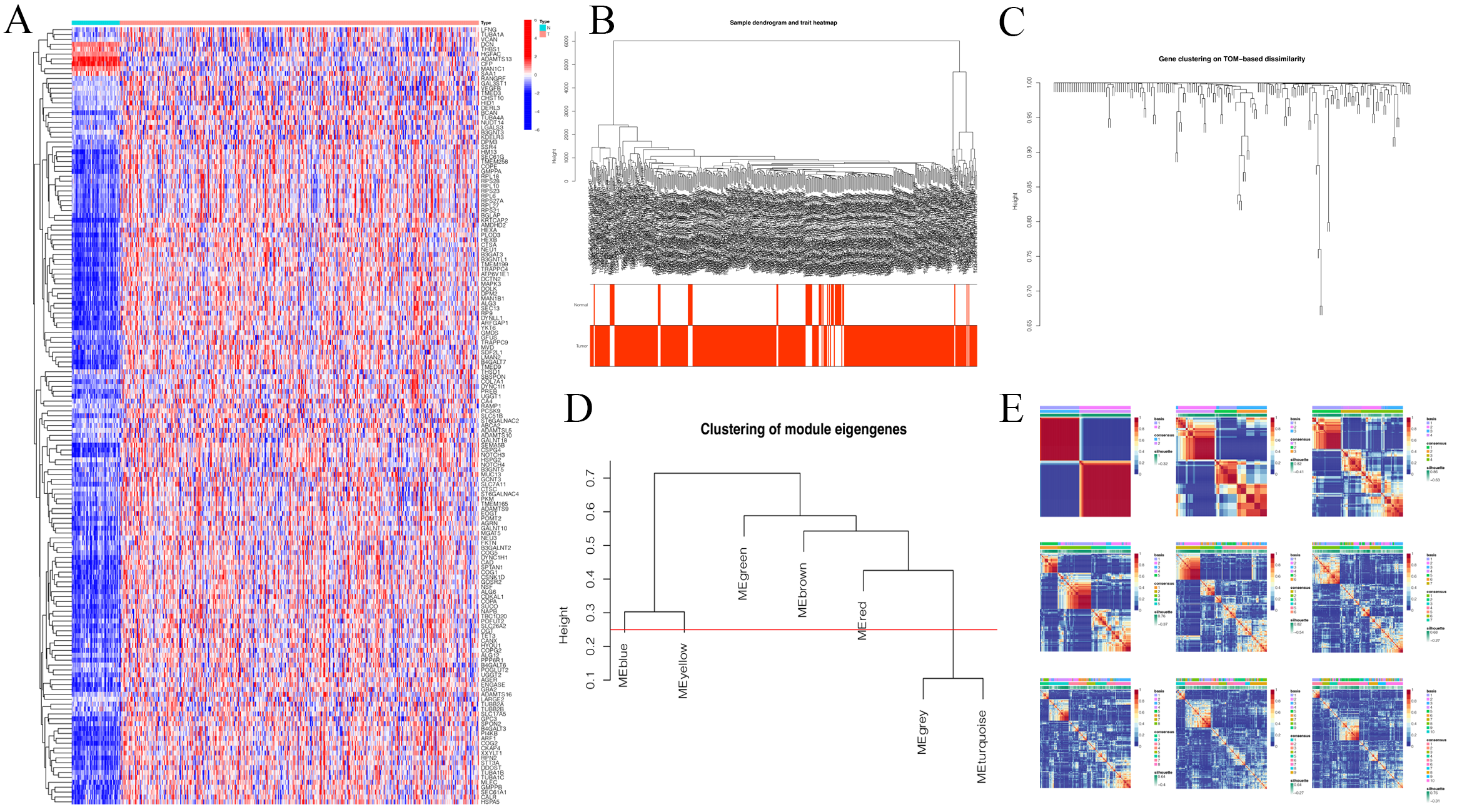

Supplement: Supplementary Figure 1 — (A) The heatmap of glycosylation-related DEGs. (B) Clustering dendrograms and trait heatmap of HCC and adjacent tissues. (C) The glycosylation-related DEGs clustering based on TOM dissimilarity. (D) All heatmaps obtained by NMF consensus clustering. [file Image_1.tif]

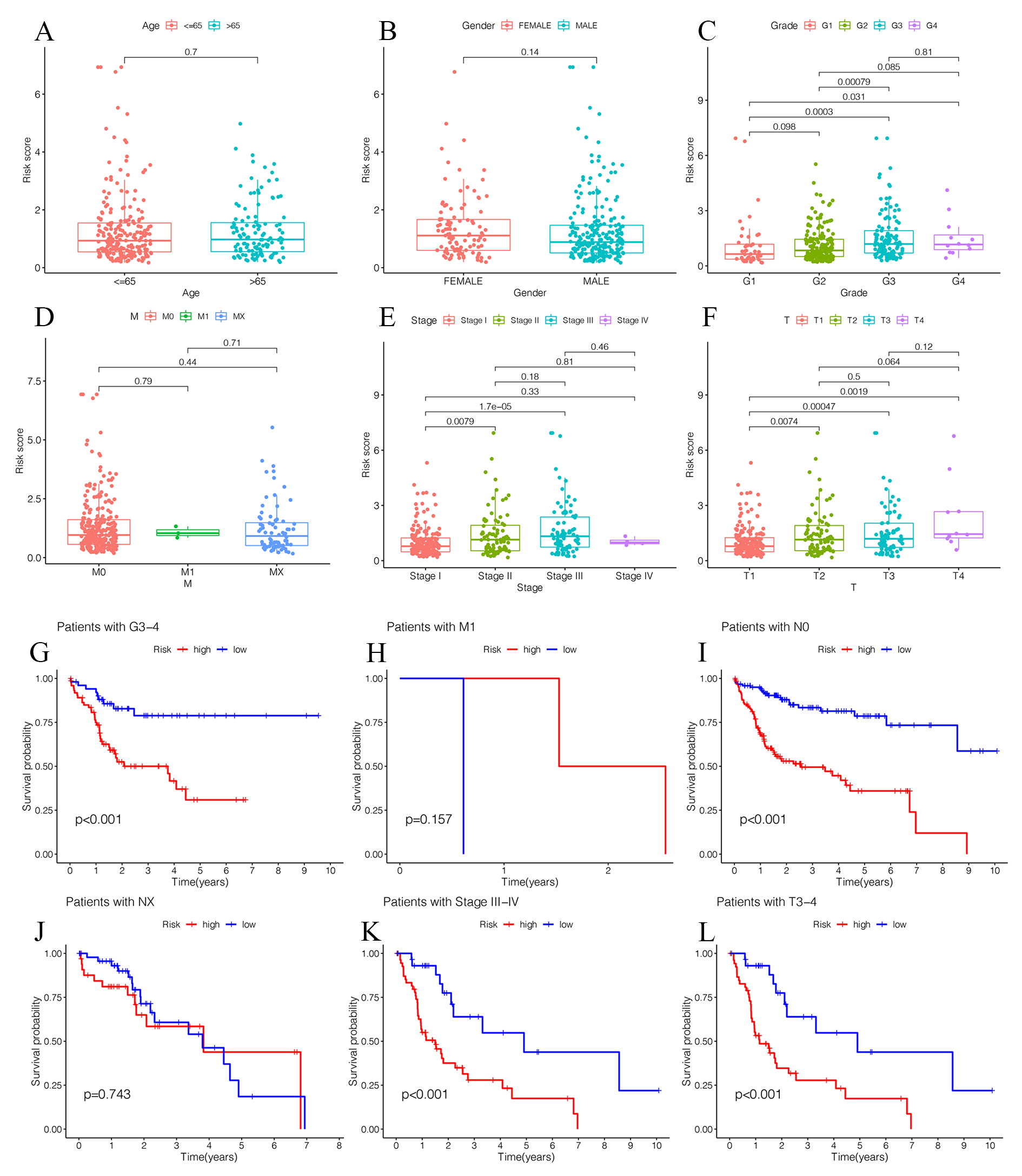

Supplement: Supplementary Figure 2 — Correlation between clinicopathological features and risk score or risk groups. Expression of clinicopathological features in risk score: (A) Age (B) Gender. (C) Grade. (D) M stage. (E) Stage. (F) T stage. Association between clinicopathological features and low- and high-risk groups by K-M survival analysis: (G) Grade 3-4. (H) M1 stage. (I) N0 stage. (J) Nx stage. (K) Stage III-IV. (L) T3-T4 stage. [file Image_2.tif]

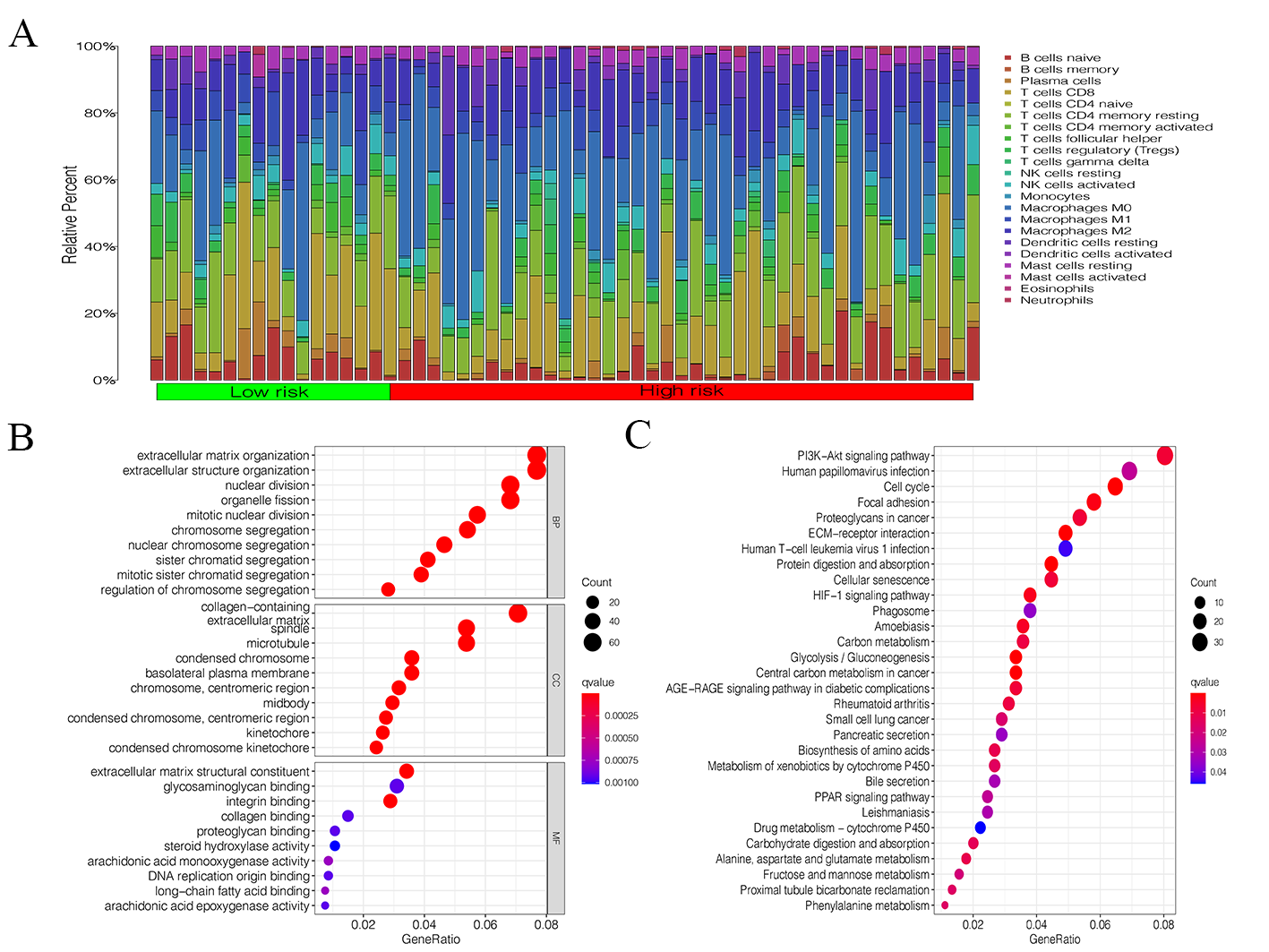

Supplement: Supplementary Figure 3 — Analysis the proportion of immune infiltration cells and functional enrichment. (A) The proportion of immune infiltration cell in low- and high-risk groups. (B) The biological functions, cellular component, and molecular function between low- and high-risk groups. (C) The mainly signaling pathway by KEGG analysis. [file Image_3.tif]
